# Current progress and prospects for G protein-coupled estrogen receptor in triple-negative breast cancer

**DOI:** 10.3389/fcell.2024.1338448

**Published:** 2024-02-27

**Authors:** Duo Zhang, Hong Chen, Jinpeng Wang, Jiale Ji, Murshid Imam, Zhijie Zhang, Shunchao Yan

**Affiliations:** Department of Oncology, Shengjing Hospital of China Medical University, Shenyang, China

**Keywords:** G protein-coupled estrogen receptor, triple-negative breast cancer, tumor development, mechanism, prognostic

## Abstract

Triple-negative breast cancer (TNBC) is a biologically and clinically heterogeneous disease. The G protein-coupled estrogen receptor (GPER) plays a crucial role in mediating the effect of estrogen and estrogen-like compounds in TNBC cells. Compared with other subtypes, GPER has a higher expression in TNBC. The GPER mechanisms have been thoroughly characterized and analyzed in estrogen receptor α (ERα) positive breast cancer, but not in TNBC. Our previous work revealed that a higher expression of GPER mRNA indicates a better prognosis for ERα-positive breast cancer; however, its effects in TNBC differ. Whether GPER could serve as a predictive prognostic marker or therapeutic target for TNBC remains unclear. In this review, we provide a detailed introduction to the subcellular localization of GPER, the different effects of various ligands, and the interactions between GPER and closely associated factors in TNBC. We focused on the internal molecular mechanisms specific to TNBC and thoroughly explored the role of GPER in promoting tumor development. We also discussed the interaction of GPER with specific cytokines and chemokines, and the relationship between GPER and immune evasion. Additionally, we discussed the feasibility of using GPER as a therapeutic target in the context of existing studies. This comprehensive review highlights the effects of GPER on TNBC, providing a framework and directions for future research.

## 1 Introduction

According to the International Agency for Research on Cancers GLOBOCAN, global cancer burden data demonstrate that breast cancer has emerged as the most commonly diagnosed cancer, accounting for roughly 2.3 million (11.7%) new cases in 2020 (Sung et al., 2021; Chhikara and Parang, 2023). Breast cancer is the primary reason for cancer-related mortality among women worldwide (Cao et al., 2021). Triple-negative breast cancer (TNBC) accounts for approximately 15% of all breast cancer cases and >50% of breast cancer-related deaths (Morris et al., 2007). This subtype of breast cancer is particularly aggressive and lacks estrogen receptor (ER), progesterone receptor (PR), and human epidermal growth factor receptor 2 (HER2).

TNBC demonstrates heterogeneity in both genetic expression and biological behavior, thereby contributing to its poor prognosis. Numerous studies have prioritized the accurate classification of TNBC to identify targeted treatments. Lehmann et al. (2011) identified seven subtypes of TNBC cell lines: luminal androgen receptor, immunomodulatory, mesenchymal stem-like, basal-like 1, basal-like 2, mesenchymal, and unstable. Moreover, Dai et al. (2017) proposed that TNBC cell lines can be divided into two distinct subtypes based on a diverse range of genetic expressions. The first subtype, triple-negative A, is characterized by an enrichment of basal markers, whereas triple-negative B is defined by a gene expression profile that indicates an increased potential for tumor invasiveness. This classification system assists in identifying high-risk individuals among patients with TNBC. Furthermore, Jiang et al. (2019) outlined a multi-omics landscape of TNBC, leading to the categorization of TNBC into four subtypes: immunomodulatory, basal-like immune-suppressed, luminal androgen receptor, and mesenchymal-like. This approach identified potential therapeutic targets for each subtype and optimized the precision treatment strategy for TNBC (Jiang et al., 2019). TNBC is resistant to specific hormone therapies and presents limited treatment options compared with those of other breast cancer types. To date, chemotherapy has been the primary approach for improving outcomes for patients with TNBC. Although new therapies such as poly (ADP-ribose) polymerase inhibitors and immunotherapy have been formulated in recent years, the TNBC prognosis remains poor due to heterogeneity (García Fernández et al., 2012; Li et al., 2017; Sharma, 2018; He et al., 2020; Yin L. et al., 2020). Therefore, identifying the molecular biological factors that impact or predict TNBC prognosis, exploring their mechanisms of action, and developing effective targeted therapeutic drugs with few side effects are crucial.

The lack of a response to hormones is regarded as an essential feature of TNBC. Nevertheless, researchers have observed the expression of certain non-classical steroid endocrine receptors in TNBC, in addition to classical ER. The G protein-coupled estrogen receptor (GPER), serving as a non-nuclear receptor, signifies the biological and clinical significance of steroid hormones in TNBC. Notably, GPER exhibits a higher expression level in TNBC than in other subtypes (Steiman et al., 2013). Existing research is controversial, with some studies indicating that GPER has a tumor-promoting effect in TNBC and is associated with increased recurrence rates, whereas others indicate the protective anti-tumor effect of GPER (Yu et al., 2014; Hernández-Silva et al., 2020). Therefore, a comprehensive perspective is required. In this review, we summarized the effects of GPER on TNBC from the perspective of signaling pathways and related mechanisms of action. Moreover, we have explored the potential of GPER as a prospective therapeutic target for TNBC based on existing original research.

## 2 G protein-coupled estrogen receptor

### 2.1 Discovery of GPER in breast cancer

**FIGURE 1:**
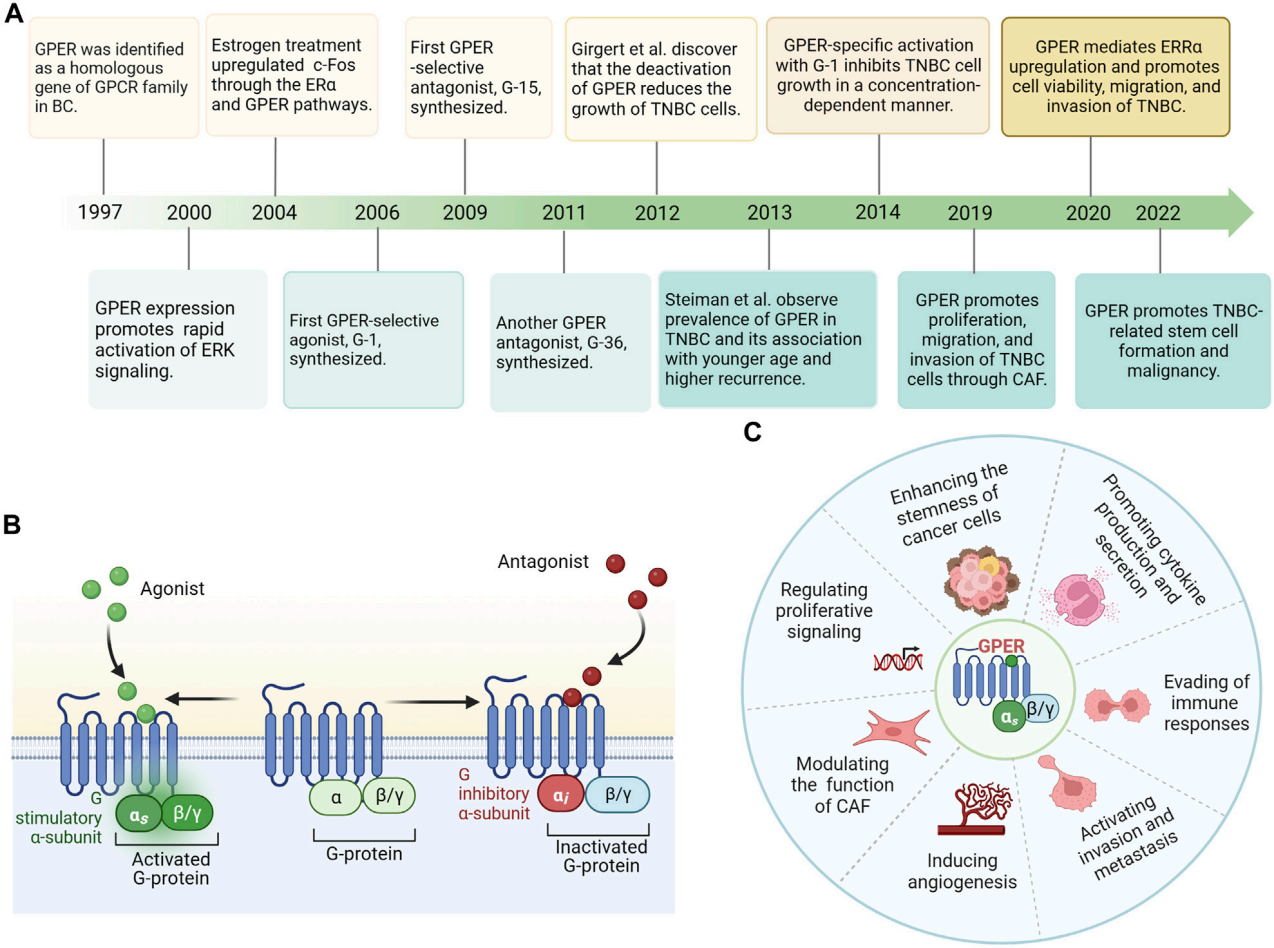
**(A)** Timeline of G protein-coupled estrogen receptor (GPER) exploration in breast cancer. **(B)** GPER protein is predominantly localized to the plasma membranes with seven transmembrane domains, ligand binding, and G protein binding pockets. Estrogen/agonist binding to GPER activates a stimulatory G protein α-subunit (Gα*_s_*) and results in GPER stimulation. Antagonist binding to GPER leads to an inhibitory G protein α-subunit (Gα*_i_*) and inactivated GPER. **(C)** GPER’s diverse functions include regulation of cell proliferation, invasion, metastasis, and angiogenesis, modulation of cancer-associated fibroblasts (CAFs), control of tumor stem cell functionality, cytokine production, and secretion, and evasion of immune responses. Abbreviations: GPER, G protein-coupled estrogen receptor; BC, breast cancer; TNBC, Triple-negative breast cancer; CAF, cancer-associated fibroblasts.

Since the 1960s, researchers have observed that the uterine adenyl cyclase system and intracellular calcium have an instantaneous ability to react with estrogen in ovariectomized rats, which initiates cyclic adenosine monophosphate (cAMP) formation and induces calcium mobilization (Szego and Davis, 1967; Pietras and Szego, 1975). These effects are referred to as extranuclear, non-genomic effects (Luo and Liu, 2020). Thereafter, Aronica et al. (1994) demonstrated that estrogen increases adenylate cyclase activity, leading to increased cAMP production in human MCF-7 cells. At this time, researchers were not aware that GPER, but not just ERα played a key role in this effect (Aronica et al., 1994). Subsequently, the GPR30 gene was identified as a gene homologous to the G protein-coupled receptor (GPCR) family in breast cancer (Carmeci et al., 1997; Levoye et al., 2006). Moreover, Filardo et al. (2000) found that the rapid activation of extracellular signal-regulated kinases (ERK) by 17β-estradiol (E2) requires the participation of an orphan GPCR, with seven transmembrane domains. Maggiolini et al. (2004) demonstrated the ability of E2 to stimulate c-Fos gene expression in both ERα-positive MCF-7 and ERα-negative SKBR3 human breast cancer cells. This effect occurred through two different pathways: one involving the ERα and the other through the GPCR homolog GPER, independent of the ERα (Maggiolini et al., 2004). Revankar et al. (2005) eventually established GPER as a distinct ERα in SKBR3 cells, with a signaling pathway different from ERα-mediated signaling. Additionally, they observed E2 as the natural ligand for GPER and found that the effects of estrogen on this receptor were likely influenced by its intracellular localization. Subsequently, specific agonists (G-1) and antagonists (G-15 and G-36) targeting GPER were sequentially developed. With the deepening of our understanding of TNBC, studies on the involvement of GPER in TNBC have gradually increased. Since 2012, attention has been focused on the relationship between GPER activation and clinical pathological characteristics in TNBC. Numerous studies have focused on further investigating the mechanisms of GPER action in estrogen signaling and its implications for the development and progression of TNBC. The exploration of GPER in breast cancer is summarized in the form of a timeline in Figure 1A.

### 2.2 Subcellular localization of GPER

As a seven-transmembrane G protein-coupled receptor, GPER is typically assumed to be located on the plasma membrane, similar to most GPCRs (Thomas et al., 2005). While GPER is concentrated in the plasma membranes of certain tissues, its intracellular detection has also been reported (Filardo et al., 2007). Furthermore, the distribution of GPER varies across species, tissues, and cell types. Initially, Funakoshi et al. (2006) observed that GPER was localized in the plasma membrane of pyramidal neurons in the hippocampal CA2 area of the rat brain. Moreover, upon agonist stimulation, the GPER-transfected HeLa cells exhibited translocation of the receptor from the plasma membrane to the cytoplasm (Funakoshi et al., 2006). Additionally, GPER is primarily located in the plasma membrane of the uterine epithelia, myometrium, and renal epithelia (Cheng et al., 2011a; Gao et al., 2011; Lindsey et al., 2011; Maiti et al., 2011). However, a significant proportion of GPER is localized within the intracellular structure. Revankar et al. (2005) conducted a study using fluorescent E2 derivatives (E2-Alexas) to examine the localization features of GPER in monkey kidney fibroblasts. Interestingly, they observed that E2-Alexas did not effectively label the plasma membrane. Instead, it is primarily bound to the endoplasmic reticulum. Furthermore, staining with E2-Alexas or antibodies also revealed colocalization in the Golgi apparatus and nuclear membrane of GPER (Revankar et al., 2005). Similarly, in a study by Brailoiu et al. (2007), GPER was observed to be distributed within the neuronal cytoplasm using immunohistochemical analysis.

GPER primarily exhibits a cytoplasmic staining pattern in breast carcinoma tissues, with minimal presence on the cell surface (Thomas et al., 2005; Filardo et al., 2006; Luo et al., 2011). Interestingly, a fraction of tumor specimens exhibited both nuclear and cytoplasmic staining (Ignatov et al., 2011; Samartzis et al., 2014). The differential subcellular localization of GPER may be explained by a dynamic change mechanism (Cheng et al., 2011b). Under ligand-independent conditions, GPER employs an endocytic trafficking mechanism through clathrin-coated vesicles, accumulates in the perinuclear compartment, and is dispersed in the cytoplasm (Cheng et al., 2011b). Based on this evidence, we speculated that the different staining patterns may reflect the dynamic and time-dependent intracellular trafficking process of GPER. The action of GPER may be regulated by the amount of receptors present at different subcellular locations.

Differences in subcellular localization may have distinct biological implications for different breast carcinoma subtypes. Filardo et al. (2006) observed that the cytoplasmic staining of GPER in breast tumor tissue was nearly two-fold higher than that in tumors without GPER expression. Moreover, Sjöström et al. (2014) conducted a study to identify whether the subcellular localization of GPER could be an independent prognostic factor and revealed a positive correlation between the overexpression of plasma membrane GPER and a high histological grade. Therefore, plasma membrane localization of GPER may be a critical event that suggests a poor prognosis for breast cancer (Sjöström et al., 2014). Samartzis et al. (2014) observed a substantial correlation between the presence of cytoplasmic GPER and low tumor stage, luminal subtypes, and improved histological differentiation. In contrast, the expression of nuclear GPER is strongly associated with the triple-negative subtype and poorly differentiated tumors (Samartzis et al., 2014). Consistent results have been reported in our meta-analysis, where high GPER cytoplasmic expression, but not nuclear expression, is associated with improved outcomes in ERα-positive breast cancer (Zhang et al., 2022). As an understudied and important potential next frontier, whether the subcellular location of GPER in breast cancer has distinct prognostic implications needs further investigation.

### 2.3 GPER ligands

Revankar et al. (2005) found that the natural hormone E2 is a paramount ligand of GPER (Thomas et al., 2005). Estriol (E3), an E2-based steroid, has been identified as a GPER antagonist, and estrone has been described as an agonist for GPER (Lappano et al., 2010). GPER exhibits a higher binding affinity to E2 than that of estrone and estriol (Revankar et al., 2005). The abundant cholesterol metabolite 27-hydroxycholesterol is a novel ligand for GPER, and its signaling axis plays a crucial role in ERα-negative breast cancer progression (Avena et al., 2022).

In 2006, researchers synthesized G-1, the first selective agonist for GPER (Bologa et al., 2006). Subsequent studies revealed a binding affinity of 10 nM between G-1 and GPER. In 2009, a selective antagonist of GPER, referred to as G-15, was shown to inhibit the calcium mobilizing effect of E2 in the SKBR3 cell line (Dennis et al., 2009). After 2 years, another GPER antagonist, G-36, was synthesized. This compound had a high affinity for GPER and a weak cross-reactivity with ERα (Dennis et al., 2011). Furthermore, Lappano et al. (2012) investigated two additional molecules, GPER-L1 and GPER-L2, which function as specific agonists of GPER, activating downstream signaling pathways.

Tamoxifen is the first accredited selective ERα modulator for breast cancer therapy and has demonstrated efficacy in reducing recurrence rates and improving the prognosis of ERα-positive breast tumors (Ariazi et al., 2006; Early Breast Cancer Trialists’ Collaborative Group, 2011). Tamoxifen acts as a GPER agonist, induces aromatase expression, and contributes to resistance to endocrine therapy in breast cancer (Meijer et al., 2006; van Agthoven et al., 2009; Ignatov et al., 2011; Catalano et al., 2014; Yin et al., 2017; Molina et al., 2020; Yu et al., 2020). Similarly, as a selective ERα downregulator, ICI182,780 (fulvestrant) displays significant binding to GPER and activates GPER in breast cancer (Osborne et al., 2004; Thomas et al., 2005).

Several plant-derived natural phytoestrogens have been shown to bind and/or activate ERα and other ERs (specifically GPER), such as daidzein (Kajta et al., 2013), genistein (Thomas and Dong, 2006; Vivacqua et al., 2006), resveratrol (Dong et al., 2013), and quercetin (Maggiolini et al., 2004). In addition, numerous xenoestrogens have been recognized as GPER ligands, including dichlorodiphenyltrichloroethane (Kajta et al., 2014), methoxychlor, atrazine (Thomas and Dong, 2006), bisphenol A (BPA) (Zhang et al., 2016), and bisphenol S (BPS) (Deng et al., 2018). The significance of BPA and BPS actions on GPER in the growth and migration of TNBC cells has been revealed. BPA is an industrially synthesized chemical compound with endocrine-disrupting effects that promote the growth of rat mammary tumor cells (Vandenberg et al., 2008; Wang et al., 2014; Zhang et al., 2016). In TNBC, BPA activates the focal adhesion kinase (FAK)/steroid receptor coactivator (SRC)/ERK signaling pathway and focal adhesion assembly by activating GPER and inducing epidermal growth factor receptor (EGFR) transactivation (Castillo Sanchez et al., 2016; Castillo-Sanchez et al., 2020). Tetrachlorobisphenol A (TCBPA) is a chlorinated derivative of BPA and exhibits estrogenic activity. Low TCBPA concentrations regulate the expression of GPER and its downstream target genes, resulting in the proliferation of TNBC cells (Lei et al., 2021). In comparison to BPA, BPS exhibits greater stability while exhibiting strong estrogenic responses. BPS was found to facilitate TNBC cell metastasis through the GPER/Hippo-Yes-associated protein 1 pathway (Zhao et al., 2008; Furth and Aylon, 2017), but it does not affect tumor proliferation (Deng et al., 2018). Deng et al. (2018) suggested that the reduced stimulation of proliferation by BPS in their study may be attributed to differences in cell lines or ERα/β expression status. Altogether, the positive and negative inhibitory effects of these GPER ligands provide a foundation for further exploration (Table 1).

**TABLE 1:** Mechanisms of GPER agonists and antagonists in breast cancer.

Name	Mechanism	Experiment cell lines	Specificity for GPER	References
Agonist				
G-1	Binds specifically to GPER and activates the GPER/EGFR/ERK signaling pathway	HCC 1806, HCC70, MDA-MB-453, SKBR3	Specific	Bologa et al. (2006)
17β-estradiol (E2)	Binds to GPER and induces rapid activation of the GPER/EGFR/ERK signaling pathway, which promotes breast cancer cell growth and invasion	MCF-7, SKBR3	Non-specific	Yu et al. (2014)
		MDA-MB-468, MDA-MB-231, MDA-MB-436		
GPER-L1	Binds specifically to GPER, thereby upregulating GPER target genes and inducing breast cancer cell proliferation	SKBR3	Specific	Lappano et al. (2012)
GPER-L2				
Tamoxifen	Binds to GPER, upregulates GPER expression, and promotes breast cancer cell proliferation, resulting in endocrine resistance	MCF-7, SKBR3	Non-specific	Catalano et al. (2014)
27-hydroxycholesterol	Binds to GPER and mediates the activation of ERK1/2 and NF-κB, thereby increasing tumor proliferation	MDA-MB-231, MDA-MB-468	Non-specific	Avena et al. (2022)
ICI182,780 (fulvestrant)	Activates the ERK and PI3K pathway through binding to GPER, resulting in endocrine resistance	MCF-7	Non-specific	Osborne et al. (2004)
Bisphenol A (BPA)	BPA activates GPER and stimulates the FAK/SRC/ERK and EGFR signaling pathway and mediates breast cancer cell migration	MDA-MB-231	Non-specific	Castillo-Sanchez et al. (2020)
tetrachlorobisphenol A (TCBPA)	TCBPA upregulates GPER expression and mediates ERK and Akt signaling, resulting in breast cancer cells proliferation	MCF-7, SKBR3, MDA-MB-231 cells	Non-specific	Lei et al. (2021)
Bisphenol S (BPS)	BPS facilitates TNBC cell metastasis through the GPER/Hippo-YAP pathway	MDA-MB-231, BT-549 cells	Non-specific	Deng et al. (2018)
Berberine (BBR)	Promotes the transcription of GPER and inhibits the viability and migration of cells	MDA-MB-231, MDA-MB-436, MDA-MB-468	Non-specific	Qi et al. (2021)
Chrysin-nanoparticles (NP)	NPs inhibit proliferation and migration of TNBC via activation of GPER and suppress PI3K, p-JNK, and NF-κB expression	MDA-MB-231	Non-specific	Kim and Jung (2020)
Tanshinone IIA	Binds to GPER and induces apoptosis in TNBC cells and inhibits migration via the GPER/EGFR/ERK signaling pathway	MDA-MB-231	Non-specific	He et al. (2023)
Antagonist				
G-15	Inhibits GPER-dependent E2 signaling	HCC 1806, HCC70	Specific	Dennis et al. (2009)
G-36	Inhibits GPER-dependent E2 signaling	SKBR3	Specific	Dennis et al. (2011)
Estriol (E3)	Inhibits the GPER/EGFR/ERK signaling pathway and breast cancer cell proliferation	SKBR3	Non-specific	Lappano et al. (2010)

Abbreviations: GPER, G-protein-coupled Estrogen Receptor; E2, estrogen; ERK, extracellular signal-regulated kinase; EGFR, epidermal growth factor receptor; FAK, focal adhesion kinase; SRC, steroid receptor coactivator; Akt, protein kinase B; TNBC, triple-negative breast cancer; YAP, Yes-associated protein 1; PI3K, phosphoinositide 3-kinase; JNK, c-Jun N-terminal kinase; NF-κB, nuclear factor kappa B.

### 2.4 Role of GPER in TNBC

In normal breast tissues, GPER is moderately expressed (Uhlén et al., 2015) and has been detected in diverse cancer cell lines and malignant tumors, such as endometrial carcinoma (He et al., 2009), ovarian cancer (Albanito et al., 2007), melanoma (Fábián et al., 2017), prostate cancer (Chan et al., 2010), thyroid cancer (Vivacqua et al., 2006), and testicular germ cell tumors (Franco et al., 2011). In invasive breast cancer, GPER exhibits a positivity rate estimated between 50% and 60%, as evidenced by immunohistochemistry (Filardo et al., 2006; Ignatov et al., 2011; Steiman et al., 2013). The expression of GPER varies among different ERα-positive breast cancer cell lines. In T47D cell lines, GPER is predominantly expressed at elevated levels in the cytoplasm, whereas in MCF-7 cells, GPER expression is primarily detected in the nucleus and is low (Samartzis et al., 2014). Moreover, within a cohort of ERα-negative breast cancer, the positivity rate of GPER exceeds 60% (Luo et al., 2011). Furthermore, in TNBC, GPER is frequently overexpressed, with rates reaching as high as 68.8% (Yu et al., 2014). Based on its expression levels, GPER holds promise as both a prognostic indicator and therapeutic target. Upon binding with an agonist, GPER is activated, playing a crucial role in the initiation and progression of tumors. Its diverse functions include the regulation of cell proliferation, invasion, metastasis, angiogenesis, modulation of cancer-associated fibroblasts (CAF), control of tumor stem cell functionality, cytokine production and secretion, and evasion of immune responses (Figures 1B,C). However, the function of GPER in TNBC and its potential effectiveness in combating highly invasive TNBC remain controversial. GPER overexpression is positively associated with metastatic capability, tumor size, HER2/neu and poor survival (Steiman et al., 2013). Using GPER-specific small interfering RNA (siRNA) knockdown, complete elimination of GPER expression was demonstrated, abolishing the stimulation of certain signaling pathways responsible for amplifying proliferation, and further substantiating the significant role of GPER in promoting the proliferation of breast cancer (Girgert et al., 2012). The potential involvement of GPER-associated signaling pathways in cell invasion and migration has also been demonstrated (Yu et al., 2014; Rigiracciolo et al., 2019; Ye et al., 2019; Yin et al., 2020). *In vivo* studies revealed that breast cancer in GPER-knockout mice resulted in diminished lung metastases compared with that in wild-type mice, indicating that animals with low or no GPER expression tend to develop less aggressive breast tumors (Marjon et al., 2014). Recent research has shown a significant correlation between GPER abundance and high-risk TNBC, characterized by G3 tumors, distant metastasis, stage III, and lymph node metastasis (Xu et al., 2022). Differing opinions suggest that GPER expression may suppress tumor initiation and progression (Weißenborn et al., 2014). GPER inhibits tumor cell growth by stimulating G2/M-phase cell cycle arrest, decreasing cyclin B expression, and promoting apoptosis in ERα-negative breast cancer cells (Wei et al., 2014). Weissenborn et al. (2017) suggested that TNBC with downregulated GPER expression has a poor outcome, which may be associated with promoter methylation changes in GPER. Additionally, TNBC models treated with GPER-specific agonists exhibited reduced distant migration ability and angiogenesis of tumor cells both *in vivo* and *in vitro*, leading to a significant reduction in lung metastasis (Chen et al., 2016).

## 3 Mechanism of GPER in TNBC

**FIGURE 2:**
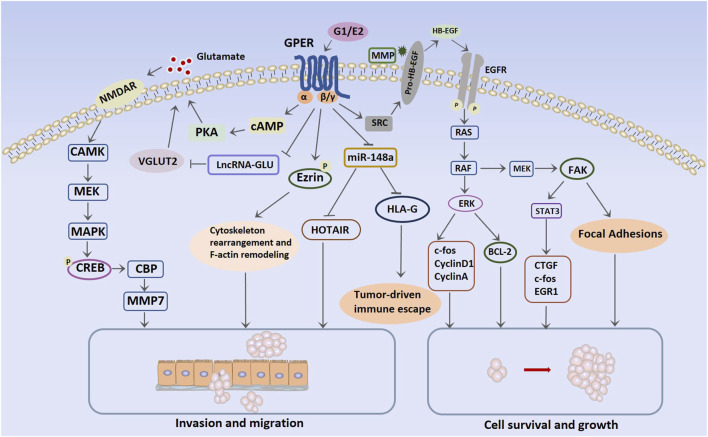
Molecular pathways mediated by G protein-coupled estrogen receptor in triple-negative breast cancer. Abbreviations: NMDAR, N-methyl-d-aspartate receptor; CAMK, Ca2+/calmodulin-dependent protein kinase; MEK, mitogen-activated protein kinase; MAPK, mitogen-activated protein kinase; cAMP, cyclic adenosine monophosphate; CREB, cAMP response element-binding protein; CBP, CREB-binding protein; VGLUT2, vesicular glutamate transporter 2; HOTAIR, HOX transcript antisense intergenic RNA; HB-EGF, heparin-binding EGF-like growth factor; BCL-2, B-cell lymphoma 2; CTGF, connective tissue growth factor; EGR1: early growth response protein 1; HLA-G, human leukocyte antigen-G; EGFR, epidermal growth factor receptor; ERK, extracellular signal-regulated kinase; FAK, focal adhesion kinase; SRC, steroid receptor coactivator.

ERs, such as ERα, belong to the nuclear receptor superfamily. E2 activates ERα in the cell nucleus, forming a dimer. The activated ERα causes gene expression in the nucleus by directly binding to estrogen response elements in the genome or by binding to other transcription factors and their response elements (Kato et al., 2005). This genomic effect is relatively slow but is an important pathway for estrogen to promote breast cancer growth (Watters et al., 1997). Additionally, a small fraction of ERα is localized to the cell membrane, enabling E2 to induce non-genomic effects through this membrane-bound ERα. Upon encountering E2 on the cell membrane, ERα rapidly activates the phosphoinositide 3-kinase (PI3K)/protein kinase B (Akt)/mammalian target of rapamycin (mTOR) pathway, which is a non-genomic effect that significantly contributes to promoting cell proliferation (Catalano et al., 2009; Puglisi et al., 2019). Furthermore, E2-ERα binding on the membrane activates the non-receptor tyrosine kinase SRC, which phosphorylates and activates aromatase, thereby promoting *de novo* synthesis of E2. This positive feedback effect also contributes to the genomic signaling of E2-ERα. Moreover, ERα signaling exhibits a ligand-independent activation pathway, exerting its effects through phosphorylation changes in ERα (Weigel and Zhang, 1998). Unlike the classic E2/ERα action pathway, E2-GPER primarily exerts its effects through fast, non-genomic signaling pathways. Distinguishing itself from ERα, which directly activates gene transcription by binding to transcriptional regulatory sites on DNA, GPER’s transcriptional effects are mediated indirectly through cAMP and EGFR. In breast cancer cell lines, E2-activated GPER couples to a trimeric G protein, and the Gα subunit dimer directly stimulates adenylyl cyclase, which in turn converts adenosine triphosphate into cAMP. In contrast, the Gβγ subunit dimer activates SRC tyrosine kinase, thereby activating α5β1 integrin and matrix metalloproteinase (MMP). Subsequently, the heparin-binding EGF-like growth factor is activated and stimulates EGFR transactivation (Filardo et al., 2000; Filardo and Thomas, 2005; Thomas et al., 2005; Filardo et al., 2008). This subsequently activates intracellular signaling events, specifically the PI3K/Akt and ERK1/2 pathways (Prossnitz et al., 2008). These two transcriptional pathways are regulated by cAMP-regulated enhancers and serum-regulated enhancers, respectively. After GPER transcriptional activation, genes for c-Fos, cyclins A, cyclins D1, and connective tissue growth factor (CTGF), which are involved in the biological processes of breast cancer, are upregulated. For example, CTGF is a cytokine that enhances the migration ability of the MDA-MB-231 cell line (Pupo et al., 2017). Moreover, in SKBR3 cells, which are ERα-negative, rapid transcriptional activation of c-Fos is induced by estradiol through activation of GPER/EGFR/mitogen-activated protein kinase (MAPK) signaling cascades (Maggiolini et al., 2004). The transcription factor c-Fos is an important invasion regulator in human mammary carcinomas, impacting cell migration, morphology, and proteolytic degradation of basal membranes (Milde-Langosch et al., 2004). The GPER mechanisms of action in TNBC and comprehensive molecular pathways are presented in Figure 2.

### 3.1 GPER-activated cell signaling and the progression of TNBC

Elevated GPER expression is directly related to a poor prognosis in TNBC (Steiman et al., 2013; Xu et al., 2022). Specifically, decreasing GPER expression with siRNA has been demonstrated to effectively inhibit the proliferation of TNBC cells (Girgert et al., 2012; 2014). In addition, G-1 has been shown to stimulate tissue proliferation by increasing mitosis (Scaling et al., 2014). Therefore, we summarized the relevant GPER mechanisms of action in TNBC cells.

#### 3.1.1 GPER/ERK non-genomic signaling

GPER plays a pivotal role in numerous types of cancer, including endometrial cancer, thyroid cancer, melanoma, ovarian cancer, and breast cancer, via non-genomic signaling events (Revankar et al., 2005; Vivacqua et al., 2006; Albanito et al., 2007). Yu et al. (2014) observed that GPER was not only highly expressed in TNBC but was also involved in the activation of estrogen-mediated non-genomic ERK signaling. Based on clinicopathological evidence, p-ERK1/2 was detected in more than three-quarters of the GPER-positive TNBC specimens. Furthermore, high levels of GPER and p-ERK1/2 have been found to be prevalent in patients with a positive lymph nodes, large tumor size, and particularly an advanced clinical stage (Yu et al., 2014).

E2 or G-1 can induce Akt or ERK activation through GPER/EGFR signaling (Albanito et al., 2007; Fujiwara et al., 2012). Interestingly, Yu et al. (2014) found that in TNBC cells, E2, tamoxifen, and G-1 induced the rapid activation of p-ERK1/2 but not p-Akt signaling. Activation of GPER/EGFR/ERK upregulated proliferation-related genes, including genes for c-Fos, cyclin A, and cyclin D1, in MDA-MB-468 cells, thereby promoting cell cycle progression and proliferation. Additionally, E2/GPER/ERK significantly protected MDA-MB-468 cells when exposed to a serum-free medium through the upregulation of B-cell lymphoma (Bcl)-2 expression. This indicates that GPER/ERK signaling contributes to cell growth and survival by increasing Bcl-2 levels in TNBC cells (Yu et al., 2014). The Na^+^/H^+^ exchanger regulatory factor 1 (NHERF1) is a target actuator of estrogen signaling and is significantly downregulated in the early stages of TNBC. Wang et al. (2017) showed that NHERF1 co-localized with GPER in MDA-MB-231 cells, and overexpression of NHERF1 induced the inhibition of ERK1/2 and Akt signaling and proliferation of TNBC. Therefore, the activation of GPER/ERK non-genomic signaling is a crucial mechanism that triggers various downstream signaling pathways associated with cell proliferation in TNBC.

#### 3.1.2 GPER/FAK transduction pathway

FAK is a cytoplasmatic protein tyrosine kinase and plays a crucial role in promoting tumor cell invasiveness, which is attributed to both kinase-independent and kinase-dependent scaffolding functions (Jean et al., 2014; Taliaferro-Smith et al., 2015; Shen et al., 2018; Rigiracciolo et al., 2021; Zhang et al., 2021). Notably, high expression of FAK has been observed in breast tumors. Increased levels of FAK in primary tumors are associated with a triple-negative phenotype, as well as invasive and metastatic breast cancer. Moreover, FAK amplification occurs during the early stages of breast tumorigenesis (Lark et al., 2005; Luo and Guan, 2010; Golubovskaya et al., 2014). Estrogen activates FAK via the GPER/c-SRC/mitogen-activated protein kinase kinase (MEK) transduction pathway, thereby mediating tumor cell proliferation and invasiveness (Rigiracciolo et al., 2019). Rigiracciolo et al. (2019) observed that GPER stimulated Y397 FAK phosphorylation and upregulated focal adhesions (FA) in invasive and metastatic TNBC. FAs are crucial subcellular structures that mediate cell adhesion to the extracellular matrix (ECM). Immunofluorescence studies have shown that GPER activation by E2 and G-1 leads to the involvement of FAK in signal transducer and activator of transcription (STAT)3 nuclear accumulation and changes in gene expression. As a critical factor in JAK/STAT signaling, STAT3 regulates tumor progression by controlling the cell cycle, apoptosis, angiogenesis, and immune evasion (Huang et al., 2010; Huynh et al., 2017). Additionally, both STAT3 and FAK participate in the regulation of GPER-mediated expression of multiple proliferation-associated genes, such as CTGF, c-Fos, and early growth response protein 1 (Joo et al., 2004; Pandey et al., 2009). Overall, estrogenic GPER signaling may promote the invasive ability of TNBC cells via FAK and STAT3.

#### 3.1.3 Long non-coding RNA

Glutamate is a key compound in cell metabolism that stimulates certain downstream molecular mechanisms to promote the invasion and migration of cancer cells through the N-methyl-d-aspartate receptor (Rzeski et al., 2002; Li et al., 2018). Compared with HER2-positive and ERα-positive breast cancer cells, glutamate is heavily secreted in TNBC. Glutamate can induce the para secretion of hypoxia-inducible factor-1α (HIF-1α) in TNBC (Briggs et al., 2016). LncRNAs are the largest and most heterogeneous RNA family among the non-coding RNAs that participate in the normal biological behavior of cells. Dysregulation of lncRNA expression is conducive to the occurrence of malignant tumors. Notably, estrogen-induced lncRNAs are associated with the development of breast cancer (Volovat et al., 2020). Under the stimulation of G-1 or E2, GPER-regulated lncRNAs are downregulated, thereby increasing the transport activity of glutamate and the transcriptional activity of vesicular glutamate transporter 2 (VGLUT2). Upregulation of VGLUT2, in conjunction with GPER-cAMP/PKA signaling, leads to increased glutamate secretion through the lncRNA–Glutamate–VGLUT2 pathway. This facilitates the phosphorylation of N-methyl D-aspartate receptor subtype 2B and activates the glutamate N-methyl-D-aspartate receptor (Zeng et al., 2019). Furthermore, Ca^2+^/calmodulin-dependent protein kinase and MEK/MAPK signaling are activated, thereby enhancing cAMP response element-binding protein (CREB) phosphorylation, resulting in the recruitment of CREB-binding protein to the promoter regions of MMP7, which participates in TNBC invasion (Yin J. et al., 2020).

Furthermore, HOX transcript antisense intergenic RNA (HOTAIR), a crucial lncRNA, can promote tumor cell progression and the survival of breast cancer stem cells (Deng et al., 2017) and serves as a predictor of adverse prognostic events in patients with breast cancer. E2-GPER increases HOTAIR levels in TNBC cells by suppressing microRNA-148a (miR-148a) expression. Several studies have documented a positive association between the expression of HOTAIR and the metastatic potential of TNBC (Deng et al., 2017; Lu et al., 2018; Sun et al., 2018). G-15 blocks the E2-induced upregulation of HOTAIR and reverses cell migration (Tao et al., 2015).

#### 3.1.4 TNBC-related stem cells

Cancer stem cells are capable of self-renewal and differentiation, which enables them to initiate tumor growth in new areas and form new tumors (Valastyan and Weinberg, 2011). In the context of basal-like breast cancer, stem cells are often detected through the low expression of cluster of differentiation (CD) 24 and high expression of CD44 (Honeth et al., 2008; Li et al., 2013; O’Conor et al., 2018). These cell subpopulations remain viable during chemotherapy or neoadjuvant endocrine therapy, implying their significance in the development of drug resistance (Creighton et al., 2009; Croker and Allan, 2012). Furthermore, TNBC have a higher abundance of therapy-resistant cancer stem cells than that of other subtypes, leading to increased mortality, treatment failure, and recurrence (Honeth et al., 2008; Li et al., 2013; Ma et al., 2014). Zhu et al. (2022) used a spheroid culture method to induce sphere formation in MDA-MB-468 cells and compared the malignant characteristics, GPER levels, and stemness-related markers between spherical and adherent cells. The results revealed increased expression of GPER and a high percentage of the CD44+/CD24-subpopulation in spheroid MDA-MB-468 cells. Furthermore, enhanced tumor metastasis was observed. This indicated a close connection between GPER expression and the enhancement of stemness in malignant tumors. Additionally, both spheroid and adherent cells exhibited increased GPER expression after E2 treatment, whereas GPER expression decreased after G-15 treatment. These effects were most apparent in spheroid cells, indicating that GPER positively regulated the proliferation, invasiveness, and colony formation of TNBC cells in response to E2 signaling and that GPER expression is associated with the display of stemness characteristics (Zhu et al., 2022).

#### 3.1.5 Ezrin proteins

Ezrin-radixin-moesin (ERM) proteins, which belong to the actin-binding family, play a vital role in bridging the gap between the plasma membrane and actin cytoskeleton (Ponuwei, 2016). ERM proteins have a significant impact on cancer cell polarity and migration by regulating cell signaling, cytoactivity, and the cytoskeleton (Clucas and Valderrama, 2014). Clinical and pathological evidence has revealed that GPER and ezrin protein expression have a positive relationship with TNBC pathological tissues. Additionally, the high co-expression of GPER/ezrin is strongly correlated with a poor patient prognosis, and a high GPER expression is linked to a poor prognosis in young patients with TNBC with elevated estrogen levels but not in patients post menopause. E2 has been reported to rapidly promote the phosphorylation of the ezrin protein through GPER or ERβ, causing ezrin-dependent rearrangement of the cytoskeleton and F-actin remodeling, which stimulates the migration and invasion of ERα-negative cancer cells. Notably, the stimulation of GPER counteracts ezrin activation and migration induced by ERβ in co-expressed GPER and ERβ cells, such as MDA-MB-231 (Zhou et al., 2016). This could be attributed to the presence of a certain degree of mutual antagonism between ERs. Consequently, high GPER expression is a risk factor for TNBC in patients with high estrogen levels and is likely associated with the GPER-mediated activation of ezrin and its downstream pathways.

### 3.2 GPER and breast cancer-associated fibroblasts

**FIGURE 3:**
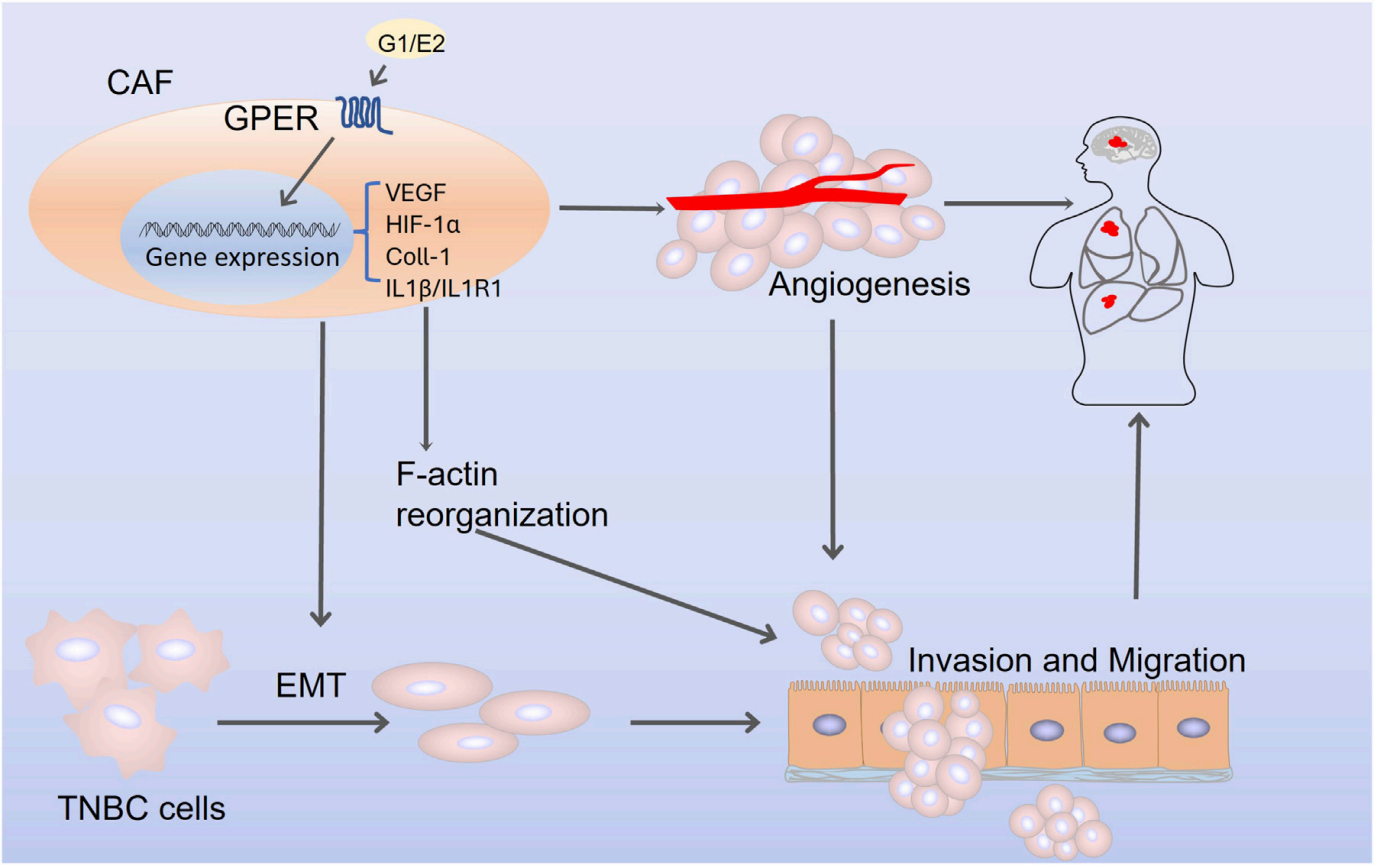
The mechanism by which G protein-coupled estrogen receptor promotes triple-negative breast cancer progression through cancer-associated fibroblasts. Abbreviations: CAF, cancer-associated fibroblasts; VEGF, vascular endothelial growth factor; EMT, epithelial-mesenchymal transition; HIF-1α, hypoxia-inducible factor-1α; Coll-1, collagen type 1; IL1β, interleukin 1β; IL1R1, interleukin 1 receptor 1.

The malignant behavior of tumors is significantly correlated with the tumor microenvironment (TME) and CAFs, which are critical members of the TME and exhibit active migratory and invasive abilities in breast cancer (Luo et al., 2015; Lappano and Maggiolini, 2018). GPER is expressed in CAFs and acts as a transcriptional regulator of the CAF response to estrogen or G-1 (Luo et al., 2014). Additionally, GPER participates in the paracrine secretion of chemotactic, growth, and ECM-related factors by CAF, thereby triggering invasive behavior in breast tumor cells (Pupo et al., 2012), including F-actin reorganization, epithelial-mesenchymal transition (EMT), cancer cell migration, and angiogenesis (De Francesco et al., 2014). Moreover, activated GPER triggers a feed-forward loop, inducing the expression of interleukin (IL) 1β/IL1R1 target genes on CAF, thereby promoting the invasive characteristics of breast cancer cells (De Marco et al., 2016). Furthermore, both estrogen and G-1 can induce the upregulation of HIF-1α and vascular endothelial growth factor (VEGF) expression in CAFs, thereby promoting tumor angiogenesis (Roskoski, 2007; De Francesco et al., 2014). In TNBC, upregulated GPER in CAF may facilitate the expression of collagen type 1 and drive the crucial proliferation, invasion, and migration of cancer cells (Yang and Yao, 2019). The above evidence demonstrates that GPER exerts a pro-tumorigenic effect on tumor cell proliferation, invasion, and migration through CAF, regardless of hormone receptor status. These mechanisms are presented in Figure 3.

### 3.3 Interaction of GPER with other hormone receptors in TNBC

The androgen receptor (AR) plays a vital role as a member of the nuclear receptor superfamily and participates in both physiological and pathological processes (Matsumoto et al., 2013; Anestis et al., 2020). Immunohistochemical staining of a TNBC tissue microarray containing 165 tumor specimens suggests that AR is positively correlated with tumor size, lymphatic metastasis, and high histological grade and negatively associated with GPER expression in TNBC (Shen et al., 2017). Dihydrotestosterone (DHT) enhances the proliferation of TNBC cells in a dose-dependent manner. Treatment with DHT leads to an upregulation of AR protein expression, whereas AR knockdown blocks the pro-proliferative effects induced by DHT. Intriguingly, DHT negatively regulates GPER expression, and AR deficiency significantly reduces the effectiveness of GPER suppression by DHT. Notably, the direct binding of AR to the GPER promoter, leading to negative regulation of GPER expression, is one of the contributing factors. The same conclusion was also observed *in vivo*, indicating that AR represses the pro-growth effects mediated by GPER in TNBC (Shen et al., 2017).

The prognostic influence of ERβ in TNBC is also disputable, with some studies suggesting that ERβ possesses anti-proliferative and -invasive functions in breast cancer cells, possibly by suppressing the PI3K/Akt/mTOR signaling pathway (Lei et al., 2020). In contrast, some studies have demonstrated that ERβ mediates TNBC invasion by regulating EGFR (Kyriakopoulou et al., 2022). In a recent study, silencing GPER expression using siRNA resulted in a weakening in the invasiveness of TNBC cells expressing ERβ while significantly enhancing the anti-invasive effects of ERβ (Schmitz et al., 2022). Activated ERβ suppresses tumor angiogenesis by downregulating VEGF protein levels (Salahuddin et al., 2022). However, GPER upregulates VEGF expression in breast cancer CAFs (De Marco et al., 2016). Additionally, in TNBC cells, the activation of GPER counteracts ezrin activation and migration induced by ERβ (Ye et al., 2019). Despite the apparent opposition in the effects of ERβ and GPER, their interaction mechanisms remain unclear and warrant further investigation.

As a classical ERα homologous orphan nuclear receptor, the overexpression of estrogen-related receptor α (ERRα) frequently correlates with unfavorable outcomes among individuals diagnosed with breast cancer (Cavallini et al., 2005; Chang et al., 2011). An *in vitro* study suggested that GPER-mediated estrogen-induced ERRα upregulation may promote TNBC cell viability, migration, and invasion. ERRα-GPER interactions lead to a malignant phenotype of TNBC, and this result was consistent with the conclusion drawn from the survival analysis (Ye et al., 2020). Additional research is necessary to determine the potential mechanisms that contribute to the interplay between GPER and ERRα in TNBC.

### 3.4 Effects of GPER on cytokines and chemokines

Activation of GPER has demonstrated effects on the synthesis of chemokines specific to white blood cells and intracellular signal transduction pathways, thereby governing the movement of immune cells and modulating inflammatory reactions. G-1 is an activator of GPER and promotes the synthesis of the anti-inflammatory cytokine IL-10 in CD4^+^ T lymphocytes (Brunsing and Prossnitz, 2011). G-1 suppresses the production of inflammatory molecules such as tumor necrosis factor (TNF), IL-6, and IL-12 in macrophages, which are triggered by lipopolysaccharides (Blasko et al., 2009). Following G-1-induced GPER activation, multiple genes, including GCSF, SOCS3, CXCL2, COX2, IL1RA, and IL1B, are upregulated through the p38 MAPK, ERK, and cAMP/PKA/CREB signaling pathways. Consequently, the production of CXCL8 protein is enhanced. Furthermore, GPER activation in neutrophils derived from healthy individuals enhanced the respiratory burst, cellular viability, and CD62L and CD11b activation marker levels (Rodenas et al., 2017).

Moreover, GPER interacts with specific chemokine receptors and cytokines, thereby impacting their signaling pathways and influencing physiological processes such as cell proliferation, migration, and tumor development. For example, the activation of GPER influences the synthesis of cytokines such as IL-6 and IL-8, which have important functions in promoting the growth and migration of cancer cells, particularly in low-oxygen environments (Bustos et al., 2017). In the case of breast cancer, the activation of GPER results in the suppression of nuclear factor kappa B (NF-κB) transcription, thereby decreasing the production of IL-6 induced by TNFα in the SKBR3 cell line. In addition, the activation of GPER is correlated with a reduction in IL-6 and VEGF-A levels in TNBC cellular models (Okamoto and Mizukami, 2016; Liang et al., 2017). Furthermore, GPER agonism by E2 inhibits TGF-β signaling in breast cancer (Kleuser et al., 2008). Both E2 and G-1 induce the release of IL-1β in CAFs, thereby resulting in an increase in the production of IL1R1. As a consequence of this molecular phenomenon, the invasive and migratory capabilities of breast cancer cells are heightened in the SKBR3 and MCF-7 cell lines. Notably, the absence of N-glycosylation in the N-terminal region of GPER causes the receptor to enter the nucleus, where it functions as a factor resembling transcription, thereby facilitating the upregulation of CTFG in CAFs and the SKBR3 cellular lineage (Pupo et al., 2017). Moreover, hypoxic conditions enhance the collaborative interaction between GPER and HIF1α in TNBC cells and CAF, thereby triggering the activation of the IL-β/IL1R1 signaling pathway and creating a feed-forward stimulatory loop. This causes changes in cytoskeleton structure and increased cellular activity, thus promoting TNBC invasiveness (Lappano et al., 2020).

### 3.5 GPER and immune evasion

GPER could mediate TNBC immune evasion and migration by regulating miR-148a in breast cancer. MiR-148a regulates tumor proliferation, invasion, and metastasis in various solid cancers by acting as an oncogene or cancer suppressor gene (Lujambio et al., 2008; Sakamoto et al., 2014). The miR-148a is specifically downregulated in TNBC (Chen et al., 2014). In their research, Tao et al. (2014) observed that estrogen-activated GPER inhibits miR-148a and stimulates the expression of human leukocyte antigen-G (HLA-G) in breast cancer (Tao et al., 2014). HLA-G is associated with the tumor-driven immune escape mechanism in the advanced stages of breast cancer (Jeong et al., 2014).

Furthermore, within the TME, the immune cells have significant contributions to tumor advancement. GPER expression is observed in diverse immune cells such as monocytes (Kvingedal and Smeland, 1997), natural killer cells (Wolfson et al., 2021), dendritic cells (Rodenas et al., 2017), macrophages (Rettew et al., 2010), polymorphonuclear cells (Blesson and Sahlin, 2012), as well as B and T lymphocytes. In breast cancer, the activation of GPER by various ligands elicits diverse responses within immune cells, influencing the constitution of the TME. Cytotoxic T lymphocytes (CTL) are the key effector cells in antitumor immunity and play an important role in immune responses against cancer. The activation of GPER leads to an elevation in IL-10 and IL-17, promoting the development of an immunosuppressive T cell phenotype. Furthermore, G-1 treatment leads to an upregulation of the programmed cell death 1 (PD-1) inhibitory receptor and CTL-associated protein 4 (CTLA-4) on isolated Foxp3+ regulatory T cells (Tregs) (Wang et al., 2009; Brunsing et al., 2013). The clinical significance of CTLA-4 and PD-1 expression in targeted cancer treatment has been acknowledged due to their ability to inhibit T cell proliferation (Zhao et al., 2018; Segovia-Mendoza et al., 2021). Thus, it is conceivable that GPER promotes the expression of PD-1 and CTLA-4 in Tregs derived from patients with breast cancer, thereby supporting the advancement of cancer. Furthermore, the use of GPER antagonists would favor the immune response against the tumor. In addition, GPER antagonists may also favor the clinical outcome of immunological checkpoints by reducing the expression of PD-1 and CTLA-4. The potential therapeutic approach of combining GPER antagonists with immunotherapy may serve as a strategy to boost the immune response and suppress the proliferation of breast cancer cells in patients.

## 4 Bi-faceted role of GPER in TNBC

Several studies have reported the antitumor functions of GPER. Our recent meta-analysis suggested that high GPER mRNA expression indicates improved overall survival in patients with ERα-positive breast cancer. Furthermore, subgroup analysis indicated that this may be related to the cytoplasmic expression of GPER (Zhang et al., 2022). Filardo et al. (2002) observed that the transfection of MDA-MB-231 cells with GPER stimulates adenylyl cyclase and inhibits EGFR-induced ERK1/2 activity (Filardo et al., 2002). Therefore, in TNBC, estrogen stimulation by GPER activation may have two different effects on the ERK1/2 pathway.

NF-κB regulates the main EMT transcription factors, including SNAIL1, SLUG, TWIST1, and SIP1, which are associated with the progression in breast cancer (Park et al., 2007; Pires et al., 2017). In TNBC, NK-κB plays an important role in the GPER-mediated inhibition of malignant tumor biological behaviors (Chen et al., 2016). Chen et al. (2016) reported that the activation of GPER by G-1 downregulates NF-κB activities and cytoskeletal protein fibronectin expression, and inhibits EMT in TNBC, resulting in a reduction in the aggressive characteristics of breast cancer. Wang et al. reported that E2-and G-1-activated GPER inhibit the expression and nuclear localization of NF-κB and STAT3 and suppress tumor angiogenesis and proliferation in MDA-MB-468 xenografts (Wang C. et al., 2018). The IL-6 cytokine family is widely expressed across vertebrates as a signaling molecule and contributes significantly to the malignant phenotype of cancer (Felcher et al., 2022). IL-6-activated STAT3 (Lee et al., 2009; Yu et al., 2009) elevates HIF-1α levels in MDA-MB-231 cells, which induces VEGF-A and MMP transcription (Xu et al., 2005; Eichten et al., 2016; Wang K. et al., 2018). Liang et al. (2017) found that the expression of IL-6 and VEGF can be suppressed by GPER-inhibiting NF-κB promoter activity in TNBC cells. Furthermore, the forceful inhibition of IL-6 expression *in vitro* reduces colony formation and cell survival and suppresses tumor engraftment in TNBC cell lines (Hanahan and Weinberg, 2011; Hartman et al., 2013; Felcher et al., 2022). Hence, activated GPER inhibits the expression of NF-κB and its downstream signaling, which are crucial in the suppression of TNBC progression. Notably, focusing on the interaction between NF-κB and GPER for the exploration of future treatments for TNBC is crucial.

Opposing evidence regarding the effect of GPER on TNBC is notable and may be associated with several factors. First, the role of G-1, as a specific ligand for GPER, in cancer cell survival and proliferation remains debatable. Further, G-1 exhibits a high affinity for GPER at a concentration of 10 nM and influences breast cancer cell proliferation in a dose-dependent manner (Table 2). Previous studies have suggested that G-1 promotes tumor cell proliferation within the range of 10 nM–1 μM, whereas high concentrations inhibit proliferation. Weißenborn et al. (2014) found that GPER treated with 1 μM G-1 inhibited TNBC cell growth. This inhibition was concentration-dependent and achieved by inducing cell cycle arrest in the G2/M phase, thereby enhancing the phosphorylation of histone h3, and promoting caspase-3-mediated apoptosis (Weißenborn et al., 2014). However, Lv et al. (2017) reported that the proliferation of TNBC cell lines was significantly inhibited by G-1 at relatively low concentrations (100 nM). G-1 inhibits microtubule assembly by occupying colchicine-binding sites within breast cancer cells, thereby inducing cell cycle arrest and triggering apoptosis. Interestingly, this inhibitory effect was not affected by GPER knockdown. Therefore, G-1 may act as a microtubule-targeting agent to inhibit breast cancer cell proliferation (Lv et al., 2017). This conclusion is similar to that of Wang et al. (2011) who demonstrated that G-1 inhibits the growth of breast and ovarian cancer cells independent of GPER at the micromolar level. In the studies mentioned above, a concentration of 1 µM was often used to assess the inhibitory effects of G-1 on breast cancer malignancy (Dennis et al., 2009; Chen et al., 2016; Liang et al., 2017). Consequently, based on this evidence, it is crucial for studies on the action of G-1 on breast cancer cells to consider both its effects on GPER and related signaling pathways and its non-GPER-dependent tumor inhibitory effect.

**TABLE 2:** Effects of different G-1 concentrations on breast cancer cell lines.

G-1 concentration	Experimental cell lines	Results	References
100 nM	MCF-7, MDA-MB-468, MDA-MB-231, MDA-MB-436	G-1 activates GPER/ERK signaling which promotes TNBC cell viability and motility	Yu et al. (2014)
1 μM	MDA-MB-231, BT-549, MDA-MB-468, HS578T	G-1 inhibited angiogenesis and migration of TNBC cells	Liang et al. (2017)
1 μM	MDAMB-231, BT-549	Treatment with G-1 for 24 or 48 h inhibited invasion of TNBC cells	Chen et al. (2016)
10^−8^–10^−5^ M	SKBR3, MDA-MB-231	G-1 inhibited the proliferation of cells in a time-dependent and concentration-dependent manner	Wei et al. (2014)
1 μM	MDA-MB-231, MCF-7, MDA-MB-468, HEK-293	G-1 inhibited the proliferation of breast cancer cells	Weißenborn et al. (2014)
500 nM, 1 μM, 2 μM	MDA-MB-231, HEK-293	G-1 suppressed TNBC cell proliferation	Wang et al. (2011)
1–10 nM, 100 nM	SKBR3, MCF-7	G-1 had no obvious effect on cell proliferation	Lv et al. (2017)
	MDA-MB-231	G-1 inhibited breast cancer cell line proliferation	
1 μM	Breast CAFs	G-1 enhanced CAF proliferation after 72-h culture	Luo et al. (2014)
1 μM	Breast CAFs, SKBR3	G-1 induced mRNA expression of VEGF.	De Francesco et al. (2014)

Abbreviations: TNBC, triple-negative breast cancer; ERK, extracellular signal-regulated kinase; CAF, cancer-associated fibroblast; VEGF, vascular endothelial growth factor.

In addition, TNBC is an extremely heterogeneous cancer that can be classified into multiple subtypes, each with a distinct gene expression profile, resulting in significant differences in treatment response and prognosis. Moreover, differences in cell types, treatment conditions, and GPER subcellular localization may contribute to the previously discussed variations. Hence, further studies are required to fully elucidate the role of GPER in TNBC.

## 5 Prospects for GPER in TNBC therapy

### 5.1 Agonists of GPER

Ligand-activated GPER can promote TNBC progression. GPER ligands mainly include endogenous estrogen, exogenous estrogen, estrogen analogs, and specific ligands. Reducing biologically available estrogen may be an effective strategy to block GPER effects. There are various methods to treat patients with ERα-positive breast cancer through blocking estrogen. These methods include ovarian removal, inhibiting ovarian function by activating gonadotrophin-releasing hormone, or using aromatase inhibitors to suppress estrogen synthesis. Investigating the specific subgroup of TNBC that could potentially benefit from estrogen deprivation strategies represents a promising avenue for future research.

With regards to the dual role of GPER in TNBC, certain GPER-specific agonists, such as G-1 could also inhibit the progression of TNBC. Berberine (BBR) is a bioactive isoquinoline alkaloid that acts as an agonist of GPER to inhibit the nuclear translocation of the NF-κB subunit RELA/p65. BBR also significantly inhibits TNBC by modulating GPER protein levels (Qi et al., 2021). The small-molecule targeted agonist of GPER, LNS8801, has demonstrated impressive results in a multicenter Phase 1–2A clinical trial, exhibiting encouraging anti-tumor effects in patients with metastatic uveal melanoma, whether used alone or in combination with pembrolizumab. Additionally, LNS8801 alone or in combination with pembrolizumab was well tolerated and did not result in unexpected toxicity (Shoushtari et al., 2023). As shown in Table 1, a series of GPER agonists have been identified, showing different mechanisms of action. Notably, limited progress has been made in the development of effective GPER agonists for the treatment of breast cancer. Chrysin is a flavone that exhibits anti-cancer effects against various types of cancers (Kim et al., 2017) including TNBC (Yang et al., 2014). Chrysin nanoparticles (NP) are polymers with improved bioavailability and water solubility. In TNBC, chrysin NPs inhibit the expression of MMPs by activating GPER and suppressing factors such as NF-κB, phosphorylated-JNK, and PI3K. MMP regulation is crucial for tumor growth, proliferation, and angiogenesis. These studies elucidate the inhibitory effects of chrysin NPs on TNBC (Kim and Jung, 2020); hence, further clinical trials are warranted.

### 5.2 Antagonists of GPER

Small-molecule antagonists of GPER mainly include G-15 and G-36. *In vitro* and *in vivo* studies have demonstrated that both have high GPER affinity and can inhibit the activity of breast tumor cells. However, there is currently a lack of supporting clinical evidence (Dennis et al., 2009; Dennis et al., 2011). Recently, DeLeon et al. (2020) discovered a novel GPER antagonist, 2-cyclohexyl-4-isopropyl-N-(4-methoxybenzyl) aniline (CIMBA), which effectively reduced estrogen-induced cholesterol gallstone formation in female mice by inhibiting GPER signaling. CIMBA exhibits fewer off-target effects than G-15 and G-36 do when binding to the receptor, and is less likely than both to bind to ERα at high concentrations. This novel antagonist also presents potential for further research in the field of cancer (DeLeon et al., 2020). As shown in Table 1, several antagonists have been identified to inhibit GPER. However, additional experimental verification is crucial to determine their efficacy in TNBC.

### 5.3 Downregulation of GPER expression

GPER, like most GPCRs, relies on the action of heterotrimeric G proteins for its signaling mechanism (Nieto Gutierrez and McDonald, 2018). Directly targeting G proteins is an effective strategy (Campbell and Smrcka, 2018). G protein inhibitors can disrupt the conformational activation of the GPCR-Gαβγ pathway. For instance, the plant-derived depsipeptide FR900359 inhibits the constitutively active G protein α subunit Gαq in uveal melanoma, thereby halting tumor cell proliferation and inducing apoptosis (Onken et al., 2020). Additionally, β-adrenergic receptor kinase 1, as a Gβγ subunit sequestering peptide, blocks the activation of ERK1/2 in breast cancer cells (Filardo et al., 2000); an effect that requires further validation in animal models. Furthermore, somavert is a competitive growth hormone receptor inhibitor and is primarily used to treat acromegaly. However, treatment with somavert suppresses the expression of GPER in TNBC cells, inhibits EGFR activation, and downregulates proliferation-associated genes, such as genes for c-Fos and cyclin D (Girgert et al., 2018). Furthermore, gene therapy techniques, such as RNA interference or clustered regularly interspaced short palindromic repeats (CRISP)-CRISPR-associated protein 9, could be used to modulate GPER expression *in vivo*. However, these methods are predominantly experimental and are not yet widely used in clinical practice.

### 5.4 Targeting the interactions between GPER and other membrane proteins

The GPER-dependent transactivation of EGFR, which plays a key role in initiating signaling pathways and influencing cellular activities, is crucial for the cellular signal transduction and function mediated by GPER (Quinn et al., 2009). Gefitinib is an inhibitor of the EGFR tyrosine kinase domain and effectively suppresses cancer cell proliferation by downregulating EGFR signaling, particularly within TNBC (Herbst et al., 2004; Bernsdorf et al., 2011). Although clinical trials using gefitinib as a monotherapy for breast cancer have exhibited unsatisfactory results, immunohistochemical analysis of post-treatment breast tumors has revealed complete inhibition of EGFR phosphorylation (Baselga et al., 2005). Girgert et al. (2017) demonstrated that gefitinib inhibits the development of TNBC cells by reducing GPER expression induced by E2. GPER antagonists efficiently impede the activation of both GPER and EGFR induced by EGF. The interplay between GPER and EGFR suggests that a therapeutic approach involving a combination of GPER and EGFR inhibitors holds promise for combating breast cancer. However, this potential requires comprehensive exploration through clinical trials.

Furthermore, the activation of the insulin-like growth factor (IGF)-1/IGF 1 receptor (IGF-1R) system induces stimulatory effects via GPER in highly malignant neoplasms such as mesothelioma and lung tumors (Avino et al., 2016). Moreover, De Marco et al. (2013) revealed a bidirectional interaction between the IGF system and GPER that promotes drug resistance and cancer progression. GPER is essential for the migratory effects induced by zinc via the IGF-1R pathway in ERα-negative breast cancer cells (Pisano et al., 2017). In addition, the fibroblast growth factor (FGF) and FGF receptor (FGFR) axis constitute a pivotal signal transduction pathway that mediates the intricate interaction between cancer cells and tumor stroma. The GPER-selective agonist G-1 induces the expression and release of FGF2 in CAFs through the activation of GPER. Subsequently, the secreted FGF2 activates the FGF2/FGFR1 paracrine signaling cascade, thereby promoting the invasion and migration of TNBC cells (Santolla et al., 2019). The interplay between GPER and these membrane growth factor receptors (GFRs) presents opportunities for the development of novel pharmacological approaches to target breast cancer progression. Therefore, combining GPER and GFR inhibitors emerges as a potential therapeutic strategy and warrants further study.

## 6 Conclusion

As a novel estrogen receptor, the biological role of GPER in TNBC has not yet been sufficiently elucidated compared with that in other breast cancer subtypes. This review presents a comprehensive overview of GPER, encompassing its subcellular structure, functional role, and internal molecular mechanisms. The analysis includes diverse ligand effects and the interactions between GPER and closely associated factors in TNBC. However, the impact of GPER on the malignant behavior of TNBC appears contradictory. Despite the existing studies, the feasibility of using GPER as a therapeutic target is insufficient, and further research is warranted.
